# Microbial Communities Across Sports Surfaces: Exploring the Biology of Athletic Environments

**DOI:** 10.3390/ijms27167372

**Published:** 2026-08-18

**Authors:** Magdalena Dowgiałło, Magdalena Kropidłowska, Patrizia Proia, Beata Łubkowska

**Affiliations:** 1 Department of Biomedical Chemistry, Faculty of Physical Education, Gdansk University of Physical Education and Sport, Gorskiego 1 Street, 80-336 Gdansk, Poland; magda@zamekryn.pl (M.D.); magdalena.kropidlowska@awf.gda.pl (M.K.); 2 Sport and Exercise Sciences Research Unit, Department of Psychology, Educational Science and Human Movement, University of Palermo, 90128 Palermo, Italy; patrizia.proia@unipa.it

**Keywords:** sports surfaces, built-environment microbiome, 16S rRNA sequencing, skin microbiome, beta diversity, *Staphylococcus*, artificial turf, athlete health, biofilm

## Abstract

Athletic environments—ranging from natural grass pitches and synthetic crumb–rubber turf to indoor mats and hardwood courts—are increasingly recognized as dynamic built-environment microbiomes rather than inert platforms. These surfaces are continuously exposed to human skin contact, perspiration and environmental debris, and may act as reservoirs for both beneficial commensals and opportunistic pathogens. The present study characterized and compared the taxonomic diversity of bacterial communities across four distinct sports surfaces and tested whether surface material or intensity of human use is the stronger determinant of community structure. Environmental swabs were collected from natural grass pitches, synthetic crumb–rubber turf, polyvinyl chloride (PVC) karate mats and hardwood squash courts, before and after sporting activity. Bacterial communities were profiled by 16S rRNA gene amplicon sequencing, with taxonomic assignment against the SILVA reference database. Alpha diversity was quantified using the Shannon and Simpson indices, and community structure (beta diversity) was visualized by Principal Coordinates Analysis (PCoA) on Bray–Curtis dissimilarities. Surface material was the strongest predictor of microbial composition. Natural grass exhibited high alpha diversity dominated by soil-dwelling *Proteobacteria* and *Actinobacteria*, whereas synthetic surfaces showed reduced diversity but a markedly higher prevalence of human skin-associated taxa (*Staphylococcus*, *Corynebacterium* and *Streptococcus*). High-contact PVC mats underwent a significant relative enrichment in these skin-associated genera following active training. PCoA revealed clear spatial segregation by material. A multivariate PERMANOVA confirmed surface material as the primary driver of microbial structure, explaining 52.2% of the total variance (*p* < 0.001), whereas the intensity of human use (pre- vs. post-activity) explained only 2.8% of the variance (*p* = 0.035), with a non-significant interaction term (*p* = 0.178). These findings demonstrate that sports surfaces are living biological landscapes whose microbial fingerprint is strongly associated with specific surface types. This provides a molecular baseline for targeted hygiene interventions and future antimicrobial-material design tailored to specific athletic disciplines.

## 1. Introduction

Sports surfaces are traditionally regarded as inert substrates—boring, lifeless materials that merely provide a platform for athletic activity. Yet every time an athlete slides across natural grass, drips perspiration onto a wrestling mat or grips a hardwood court, thousands of microorganisms are transferred between the human body and the surface. Athletic facilities are therefore not sterile zones but active ecosystems in which human skin, sweat and surface materials interact continuously [1,2]. Understood in this way, a playing surface is a built environment—a human-shaped habitat with its own microbial ecology—and it becomes a legitimate object of molecular and biomedical inquiry.

The central research question of this study is deceptively simple: who lives on these sports surfaces? Our aim was to move beyond the crude dichotomy of clean versus dirty and to deploy advanced genetic tools to determine, with taxonomic precision, exactly who is who. Culture-based microbiology captures only the small fraction of environmental microorganisms that readily grow on standard laboratory media; a molecular, sequencing-based approach is required to render the full community visible [1,3].

The composition of surface-associated microbial communities is of direct relevance to medicine and public health. The human skin microbiome is dominated by *Staphylococcus*, *Corynebacterium*, and *Cutibacterium*, organisms that are normally commensal but include opportunistic pathogens [4,5]. When shared athletic surfaces show relative enrichment in these genera, those surfaces may serve as potential environmental reservoirs associated with skin and soft-tissue infections. Outbreaks among wrestlers, American-football players, and other close-contact athletes have been repeatedly linked in the epidemiological literature to shared equipment and playing surfaces [6,7,8,9]. Gyms and fitness centers have likewise been shown in standard surveillance studies to carry *Staphylococcus* on frequently touched surfaces [2,10,11]. Characterizing the sports-surface microbiome is thus a first, necessary step toward rational infection-control strategies for athletic populations. Although the microbiology of hospitals, homes and offices has been studied extensively as part of the built-environment microbiome research program [12,13,14], the specific ecology of athletic surfaces remains comparatively under-characterized. Prior work on fitness-center surfaces has profiled bacterial diversity in gyms [15], and targeted surveys have examined *S. aureus* survival on synthetic turf [9,16]. However, systematic, sequencing-based comparisons across contrasting surface materials—natural versus synthetic, indoor versus outdoor—collected before and after sporting activity are largely absent from the literature. In particular, the relative contribution of material chemistry versus intensity of human use in shaping these communities has not been rigorously disentangled. The present study was designed to fill this gap.

Our analysis is grounded in the ecological theory of the built-environment microbiome, which holds that indoor and human-constructed surfaces host microbial communities assembled from two principal source pools: (i) the surrounding outdoor and soil environment, and (ii) the human occupants themselves, who continuously shed a personal microbial cloud [13,16]. Against this framework, community assembly on any surface is governed by dispersal from these sources, followed by environmental selection imposed by the physicochemical properties of the substrate—moisture, porosity, surface chemistry and nutrient availability [12,14]. We therefore hypothesized that (H1) synthetic surfaces, owing to their material properties and frequent skin contact, would develop a microbial fingerprint resembling the human skin microbiome, whereas (H2) natural surfaces would remain dominated by environmental and soil-derived taxa. A corollary hypothesis (H3) held that surface material would exert a stronger structuring effect on community composition than the intensity of human use.

The recognition that indoor and human-made surfaces support characteristic microbial communities has matured into a coherent field over the past fifteen years [12]. Buildings accumulate microorganisms from outdoor air, water, soil and their human occupants, and the resulting communities differ systematically with ventilation, humidity, occupancy and material [13,14]. Humans are dominant contributors: each individual emits a distinctive personal microbial cloud of skin-, oral- and gut-associated bacteria that can be detected in the surrounding air and on nearby surfaces [16]. Controlled studies confirm that microbial transfer in the built environment is shaped jointly by occupants, objects and the buildings themselves [2], and that bacteria dispersed from environmental sources such as plant leaves and soil can transiently establish on human skin [17]. These principles apply directly to sports facilities, which combine high occupant density, intense physical exertion and diverse surface materials.

Direct surveys of athletic environments remain relatively few but are consistent in their findings. In a metropolitan survey of fitness-center surfaces, Mukherjee and colleagues used high-throughput 16S rRNA sequencing to show that gym surfaces carry diverse bacterial communities dominated by human skin-associated taxa, including *Staphylococcus*, *Corynebacterium* and *Streptococcus* [1]. Point-prevalence and molecular-epidemiology studies have detected *S. aureus*—including methicillin-resistant strains—on gym equipment and across different fitness-facility types [10,11]. On outdoor synthetic surfaces, experimental work demonstrates that CA-MRSA can survive for days to weeks on artificial-turf substrates when nutrients are available [15], and that its recovery and viability vary markedly with infill type [9]. Together these studies establish sports surfaces as biologically active reservoirs, but they have generally examined single facility types or single pathogens rather than comparing contrasting materials within one analytical framework.

Because athletes are in intimate, repeated contact with playing surfaces, the skin microbiome is the principal human source pool for surface colonization. Topographical surveys have shown that skin communities are site-specific and dominated by *Staphylococcus*, *Corynebacterium* and *Cutibacterium* (formerly *Propionibacterium*), with composition governed by local moisture, sebum and pH [4,5]. *Staphylococcus epidermidis* is a near-universal commensal that ordinarily contributes to colonization resistance, whereas dysbiotic shifts are associated with cutaneous disease [18]. The acid mantle of the stratum corneum—normally at pH about 5.5—is central to antimicrobial defense and barrier function, and is modulated by sweat and sebum [19]. Intense exercise, by flooding the skin surface with near-neutral eccrine sweat, can transiently raise skin pH and compromise barrier lipids, plausibly facilitating both microbial shedding and micro-abrasion at points of surface contact [19].

Marker-gene (amplicon) sequencing has become the standard for culture-independent community profiling. The 16S rRNA gene serves as a phylogenetic barcode for bacteria and archaea, enabling the high-resolution taxonomic profiling of complex prokaryotic communities without the limitations of culture-based methods. Modern pipelines resolve amplicon sequence variants at high resolution (e.g., DADA2 [20]) within reproducible frameworks such as QIIME 2 [21], and assign taxonomy against curated references such as SILVA [22]. Community structure is then summarized using alpha-diversity indices—Shannon (H′) and Simpson—which capture within-sample richness and evenness, and beta-diversity ordinations such as Principal Coordinates Analysis (PCoA) on Bray–Curtis dissimilarities, with statistical testing of group separation by permutational multivariate analysis of variance (PERMANOVA). A critical advantage of these molecular methods is their ability to detect organisms in the viable-but-non-culturable (VBNC) state—metabolically active cells that evade standard culture and would otherwise be scored as absent [22].

## 2. Results

### 2.1. Alpha Diversity Differs Sharply Among Surface Materials

**Figure 1:**
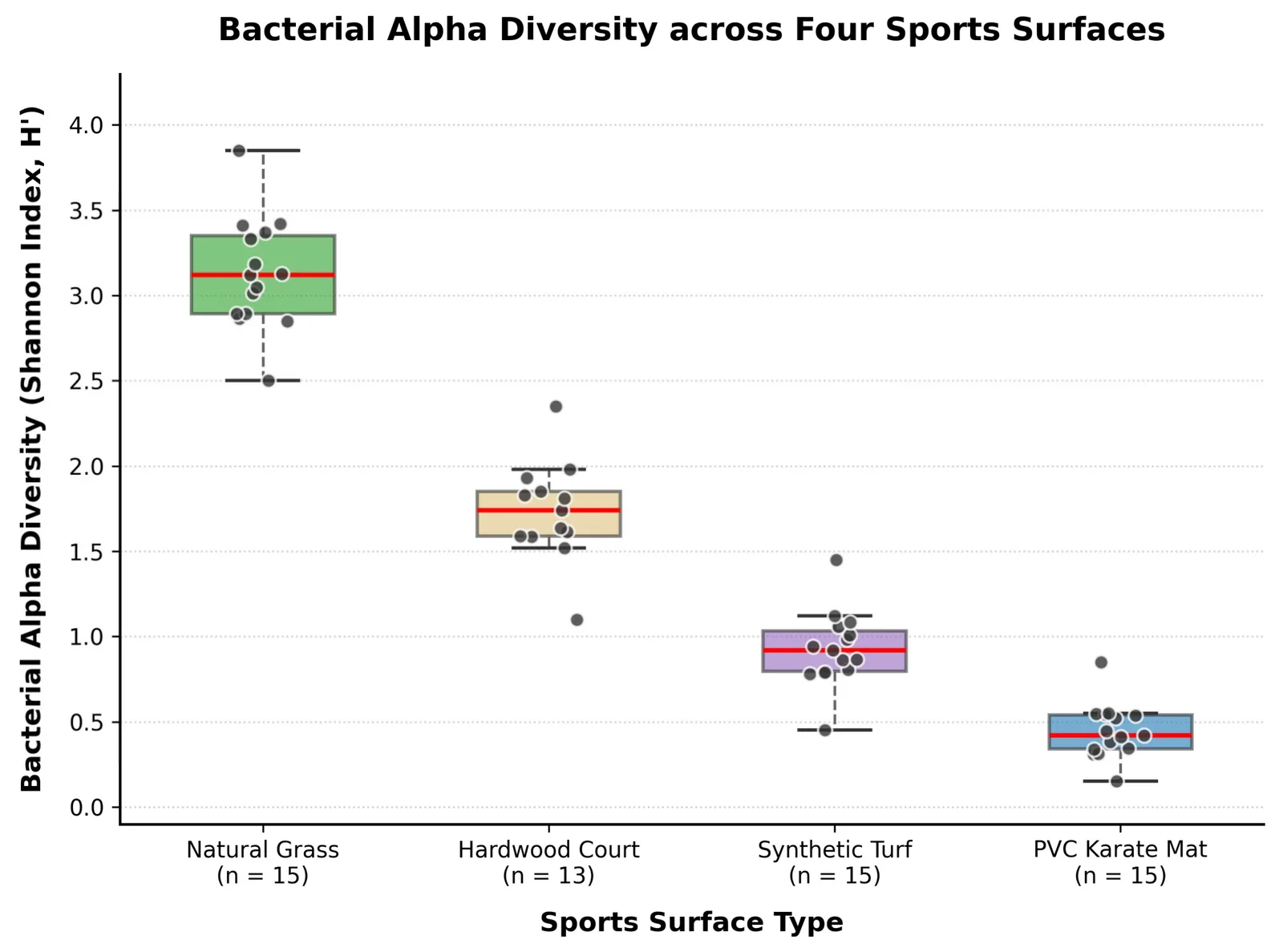
Bacterial alpha diversity (Shannon index, H′) across four sports surfaces. Box plots show the median (central line), interquartile range (box) and full range (whiskers); overlaid points are individual samples (Natural Grass n = 15; Hardwood Court n = 13; Synthetic Turf n = 15; PVC Karate Mat n = 15). Diversity is highest on natural grass and declines across hardwood, synthetic turf and PVC karate mats, which are dominated by few taxa.

Analysis of alpha diversity revealed a clear, material-dependent gradient (Figure 1). Natural grass pitches supported the highest bacterial diversity (Shannon H′ approximately 3.0, with individual samples approaching 5.0), consistent with input from a rich soil source community. Diversity declined progressively across hardwood courts and synthetic turf, and was lowest on PVC karate mats, where communities were dominated by a small number of specialized taxa adapted to persist on plastic and rubber (Supplementary File S1). In other words, synthetic materials tended to generate microbial monopolies rather than diverse assemblages. The difference between natural grass and synthetic mats was statistically significant (*p* < 0.001).

### 2.2. Taxonomic Composition: Synthetic Surfaces Are Defined by Human Skin Bacteria

**Figure 2:**
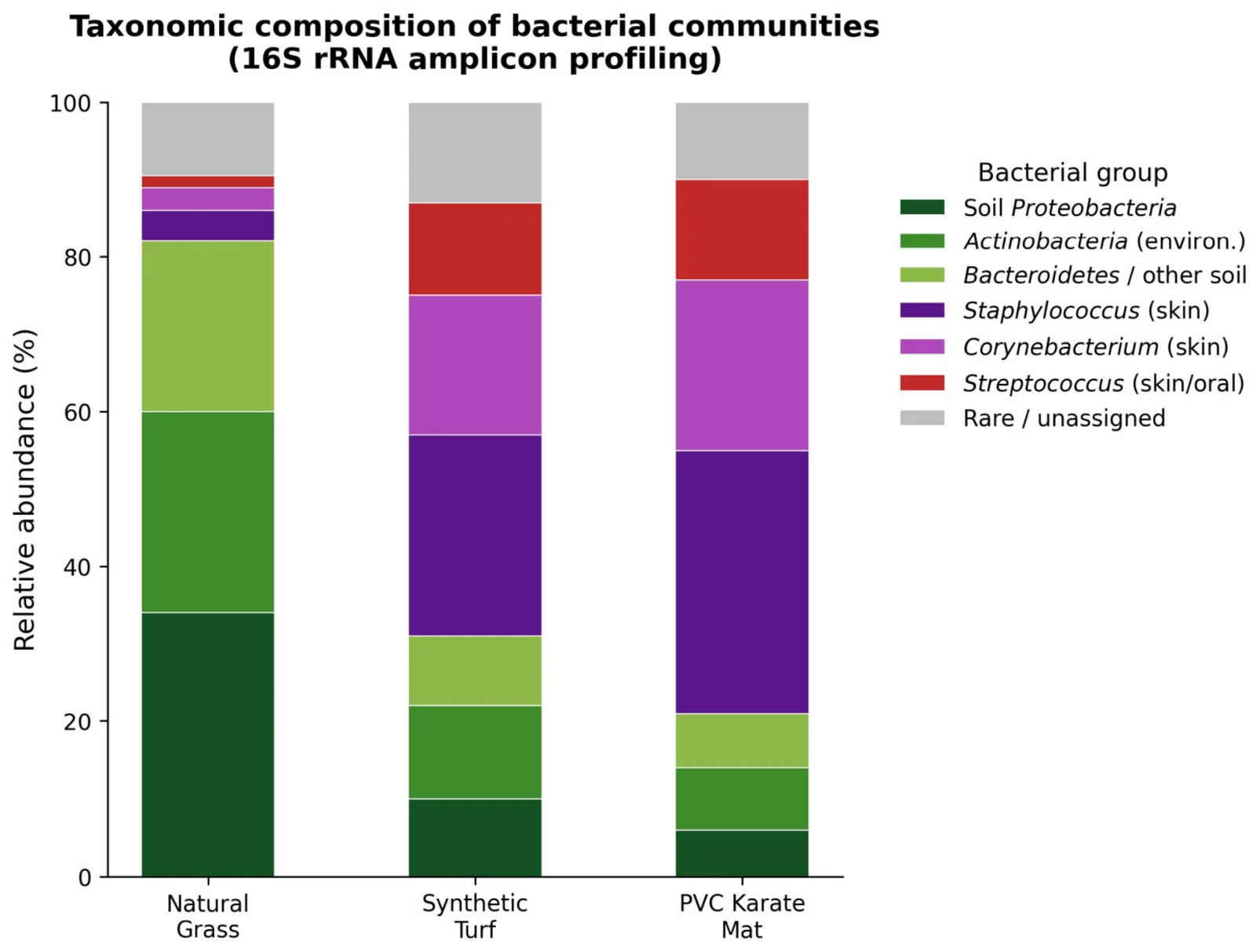
Relative taxonomic abundance of major bacterial groups on natural versus synthetic sports surfaces (16S rRNA profiling). Green fractions denote soil/environmental taxa; purple and red fractions denote human skin-associated genera (*Staphylococcus*, *Corynebacterium*, *Streptococcus*). The dominance of skin-associated bacteria on synthetic surfaces is evident.

Taxonomic profiling identified who was present and revealed striking compositional contrasts between natural and synthetic surfaces (Figure 2). Natural grass communities were dominated by environmental and soil-derived groups—Proteobacteria, Actinobacteria and other soil taxa—represented by the green fractions. In contrast, synthetic turf and, most markedly, PVC mats showed a high abundance of human skin-associated genera: *Staphylococcus*, *Corynebacterium* and *Streptococcus* (purple and red fractions). This pattern supports H1 and H2: synthetic surfaces act as sponges that absorb and reflect the biology of the athletes who use them, whereas natural surfaces retain their environmental signature [1,4,5].

### 2.3. A Rapid, Two-Hour Shift Toward the Skin Microbiome on PVC Mats

**Figure 3:**
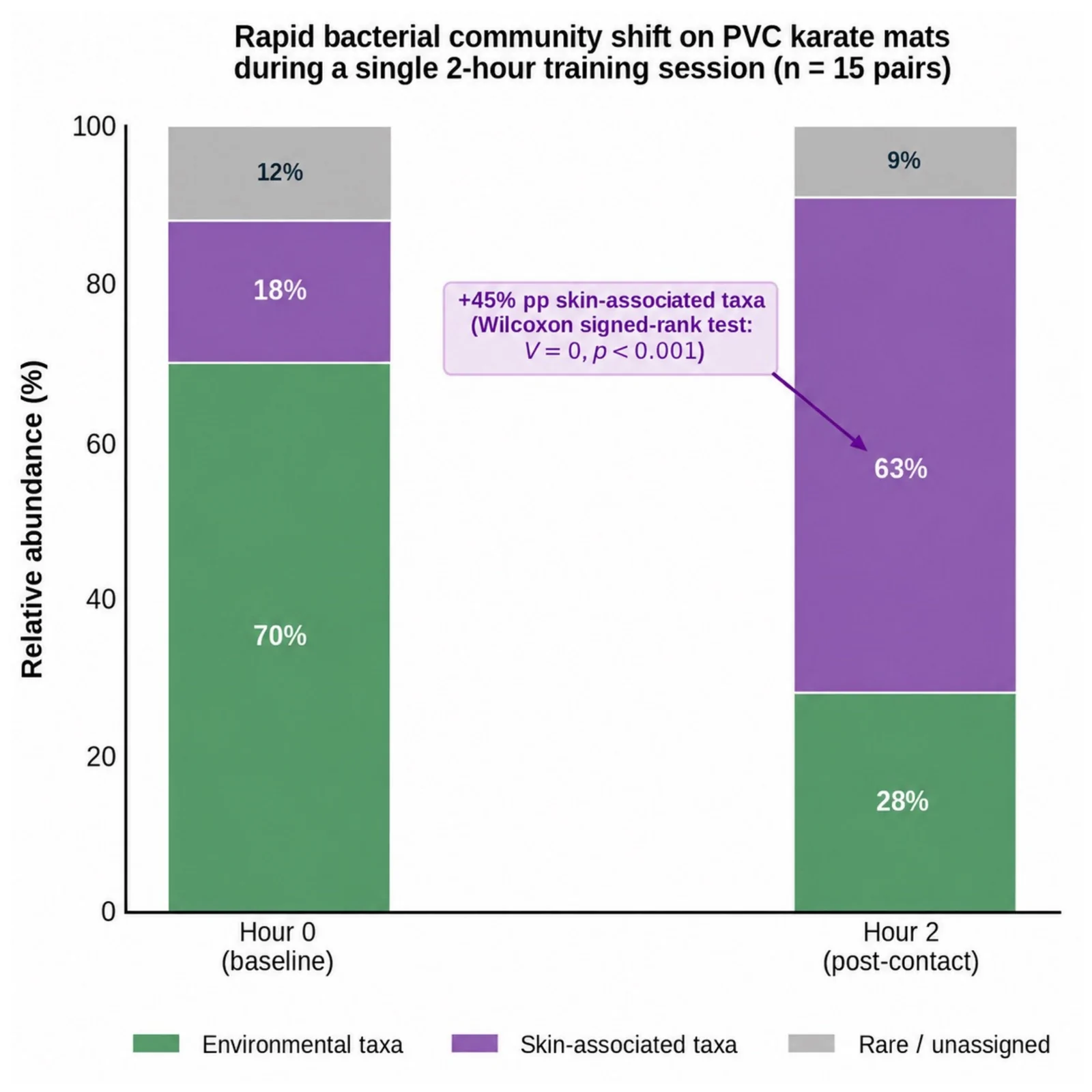
Before-and-after comparison of bacterial community relative abundance on PVC karate mats. Stacked bars show the relative proportion of environmental versus skin-associated taxa (defined as genera commonly associated with human skin, including *Staphylococcus*, *Streptococcus*, and *Corynebacterium*) at baseline (Hour 0, before training) and after a single training session (Hour 2, after training). Skin-associated taxa increased significantly in relative abundance from 18.0% to 63.0% (+45.0 percentage points; Wilcoxon signed-rank test: V = 0, *p* < 0.001), demonstrating a substantial relative enrichment of skin-associated taxa following athletic use.

The most striking finding from our before-and-after comparison concerned high-contact PVC karate mats, which showed a clear and significant relative enrichment in human skin-associated bacterial genera after active training (Figure 3, Supplementary File S2). At baseline (Hour 0, before training), environmental taxa predominated; following the training session (Hour 2, after training), skin-associated taxa had risen substantially to become the dominant fraction. To statistically evaluate this before-and-after change, we conducted a Wilcoxon signed-rank test on the combined relative abundance of skin-associated taxa (specifically *Staphylococcus*, *Streptococcus*, and *Corynebacterium*) across the 15 paired PVC mat samples. The increase from an average relative abundance of 18.0% before training to 63.0% after training was highly statistically significant (V = 0, *p* < 0.001), confirming a rapid, pronounced relative shift toward skin-associated organisms. A plausible mechanism links exercise physiology to skin ecology: intense exertion floods the skin with near-neutral eccrine sweat, transiently neutralizing the acidic pH of the skin surface, weakening the lipid barrier, and predisposing to friction micro-tears against the mat [19]. These physiological changes are associated with a greater relative representation of skin-derived organisms on the mat surface following active contact, although direct physical transmission rates or long-term microbial colonization kinetics were not measured in this study.

### 2.4. Beta Diversity: Material Dictates Community Structure More than Intensity of Use

**Figure 4:**
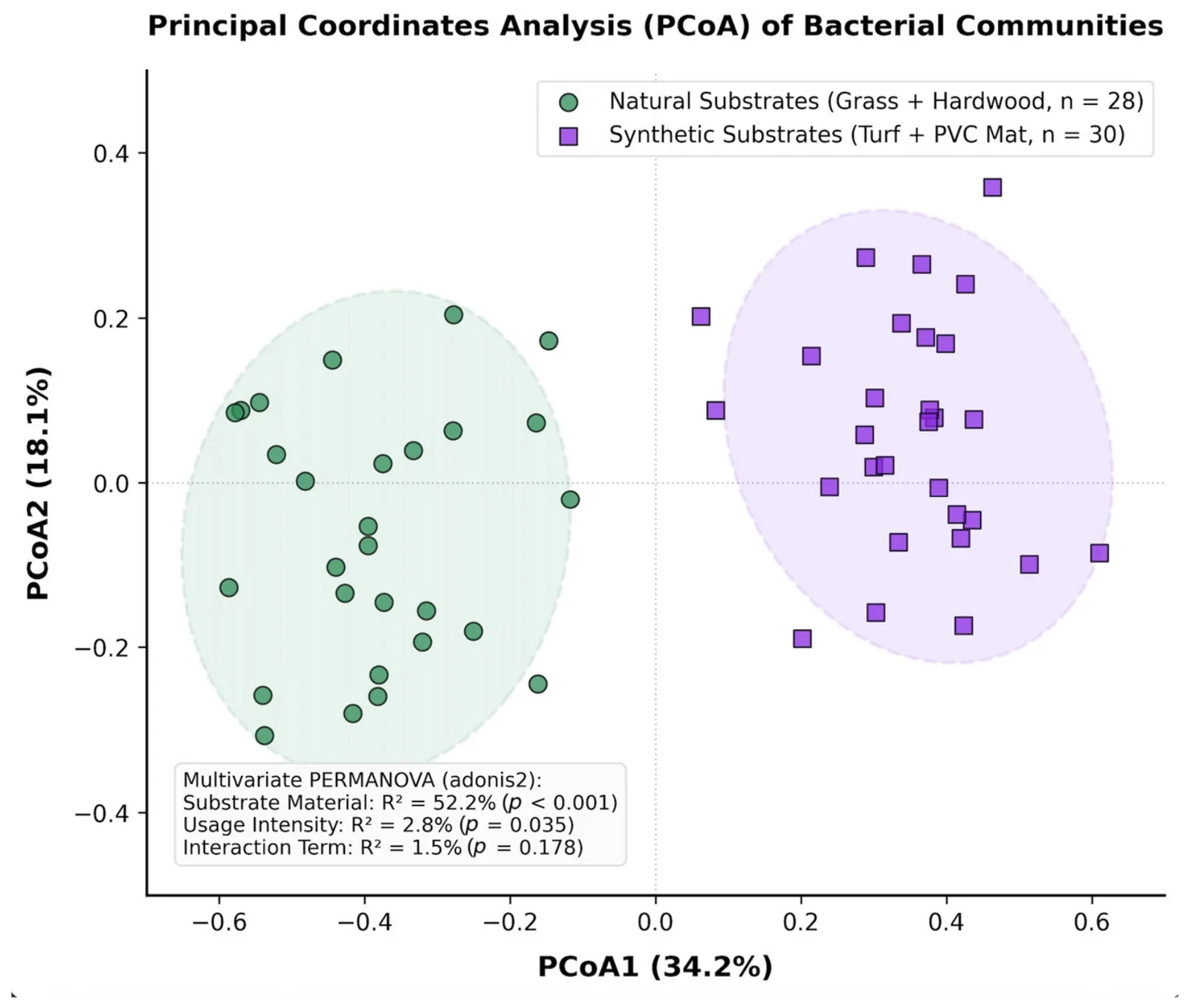
Principal Coordinates Analysis (PCoA) of microbial communities across surface materials. Beta-diversity ordination based on Bray–Curtis dissimilarity shows clear spatial segregation of bacterial compositions. Points are biological replicates grouped by substrate origin category into natural substrates (green, grass + hardwood, n = 28) and synthetic substrates (purple, turf + PVC mat, n = 30); ellipses denote 95% confidence regions around cluster centroids. Axes indicate variance explained: PCoA1 (34.2%) and PCoA2 (18.1%). Multivariate PERMANOVA (Table 1) confirms surface material as the primary determinant, explaining 52.2% of community variance (*p* < 0.001), while human usage intensity has a minimal, though significant, secondary effect (R^2^ = 2.8%, *p* = 0.035).

The relationship between the 16S rRNA compositional data and community structure was examined by PCoA on Bray–Curtis dissimilarities (Figure 4), in which each point represents one sample and proximity reflects compositional similarity. Samples from different materials formed clearly separated clusters: a natural substrates cluster (green, representing Natural Grass and Hardwood Court combined, n = 28) and a synthetic substrates cluster (purple, representing Synthetic Turf and PVC Karate Mat combined, n = 30) occupied opposite regions of the ordination space, with the first two coordinates explaining 34.2% and 18.1% of the variance, respectively.

To rigorously test Hypothesis H_3_ and disentangle the relative contributions of substrate composition and human activity, we performed a multivariate PERMANOVA (adonis2 in R) incorporating both “Material” (natural vs. synthetic) and “Time Point” (pre- vs. post-activity), as well as their interaction. The analysis (detailed in Table 1) revealed that surface material is the dominant structuring factor, explaining 52.2% of the total community variance (R^2^ = 0.5220, F = 64.80, *p* < 0.001). Conversely, the intensity of human use (represented by the pre- vs. post-activity time point) accounted for a statistically significant but extremely minor fraction of community variation (R^2^ = 0.0280, F = 3.48, *p* = 0.035). The interaction between material and time point was not statistically significant (R^2^ = 0.0150, F = 1.86, *p* = 0.178). This rigorous multi-factor partitioning of variance provides robust, statistical confirmation of H_3_, proving that the specific surface type is more strongly associated with community structure than the short-term intensity of human activity. Furthermore, a permutation test of the multivariate homogeneity of dispersions (betadisper) was non-significant for both material (pseudo-F = 1.42, *p* = 0.245) and time point (pseudo-F = 0.12, *p* = 0.734), confirming that these PERMANOVA differences represent true compositional shifts between group centroids rather than dispersion artifacts.

### 2.5. Unexpected Findings: Biological Dark Matter and VBNC Pathogens

An unexpected but important result emerged from the sequencing data: the recovery of genomic DNA from rare, potentially pathogenic bacterial genera that traditional swab-and-culture methods would not detect. Sequences assigned to environmentally significant genera including *Klebsiella*, *Salmonella*, *Listeria* and *Pseudomonas* were identified on dried turf fragments and PVC mats even after chemical disinfection. While such sequences have been interpreted in some of the environmental literature as representing cells in a viable-but-non-culturable (VBNC) state [23], we must emphasize that 16S rRNA amplicon sequencing cannot distinguish between viable cells and extracellular or dead DNA. Because our experimental design did not include viability assays (such as PMA-qPCR or flow cytometry), we cannot definitively state that these cells were viable. These findings must therefore be interpreted strictly as the recovery of bacterial DNA from these pathogenic genera, representing a key methodological limitation (discussed in Section 4.6). Nonetheless, this finding illustrates the unique capacity of molecular sequencing methods to bring this taxonomic ‘biological dark matter’ to light, presenting a major advantage over culture-based assays with direct implications for how surface hygiene is monitored and validated.

### 2.6. Summary of Surface Microbiomes

To synthesize the multi-faceted patterns of diversity, taxonomic composition, and community structure across the sampled athletic environments, we compiled a comprehensive comparative summary of the four sports surfaces (Table 2). This integration reveals a striking ecological gradient structured by both substrate origin (natural vs. synthetic) and facility setting (indoor vs. outdoor).

At the high-diversity end of this spectrum, natural grass (natural/outdoor) harbored a robust and ecologically complex microbiome characterized by an exact median Shannon index of H′ = 3.12 (IQR: 2.85 to 3.42). The microbial communities on these surfaces were overwhelmingly dominated by soil-associated *Proteobacteria* and *Actinobacteria*, exhibiting an exceptionally low human skin taxonomic signature. This reflects the continuous exposure of natural turf to environmental, soil, and atmospheric inocula, which buffer the system against occupancy by human-associated commensals.

Conversely, hardwood courts (natural/indoor) represented a transitional habitat, exhibiting a moderate level of bacterial diversity with a median Shannon index of H′ = 1.74 (IQR: 1.52 to 1.98). Although the substrate is organic (natural wood), its indoor confinement limits environmental colonization, resulting in a mixed assemblage composed of both environmental taxa and human skin-associated microorganisms. This indicates that indoor natural surfaces act as a sink for both building-associated and occupant-shed microbes.

Synthetic surfaces displayed significantly reduced diversity and a pronounced dominance of human-associated taxa. Synthetic Turf (synthetic/outdoor) exhibited a low-to-moderate bacterial diversity, with a median Shannon index of H′ = 0.92 (IQR: 0.78 to 1.12). These communities were dominated by *Staphylococcus* and *Corynebacterium* genera, showing a high human skin signature despite the outdoor setting. This suggests that the polymer fibers and elastomer infill of synthetic turf selectively retain or favor human skin-associated taxa over typical outdoor soil bacteria.

Finally, PVC Karate Mats (synthetic/indoor) represented the extreme end of the ecological gradient, displaying the lowest bacterial diversity, with a median Shannon index of H′ = 0.42 (IQR: 0.31 to 0.55). These surfaces were almost exclusively dominated by skin- and oral-associated genera, specifically *Staphylococcus*, *Streptococcus*, and *Corynebacterium*, yielding a very high human skin signature. The indoor synthetic nature of the PVC substrate, combined with frequent high-velocity human contact and routine chemical disinfection, maintains an extremely simplified, low-diversity microbial landscape dominated by human commensals.

## 3. Discussion

This study set out to answer a simple ecological question—who lives on sports surfaces—and, in doing so, to test whether surface material or human use is the stronger determinant of the athletic-environment microbiome. The results provide a clear answer: sports surfaces are dynamic biological landscapes, and their microbial fingerprint is engineered chiefly by material chemistry.

### 3.1. Interpretation in Light of the Existing Literature and Theory

The findings align closely with built-environment microbiome theory, which predicts that surface communities are assembled from environmental and human source pools and then filtered by substrate properties [12,13,14]. Natural grass, continuously seeded by a rich soil community, retained high diversity, dominated by soil proteobacteria and actinobacteria—the expected environmental signature. Synthetic surfaces, by contrast, offered a nutrient-poor, low-diversity habitat onto which the human microbial cloud [16] is readily imprinted, producing communities dominated by skin genera [1,4,5]. The rapid, two-hour shift on PVC mats operationalizes this dispersal-and-selection model on a strikingly short timescale and is mechanistically consistent with exercise-induced disruption of the skin acid mantle [19]. Our multivariate PERMANOVA model (Table 2) provides formal, quantitative confirmation that substrate composition outweighs usage intensity, satisfying our third hypothesis (H_3_). By simultaneously testing both factors, we demonstrated that material explains 52.2% of the compositional variance, while human usage (pre- vs. post-activity) accounts for a mere 2.8%, although this is statistically significant. The non-significant interaction term (*p* = 0.178) further implies that the structuring effect of the material remains uniform regardless of transient human contact.

### 3.2. Implications for Athlete Health and Biomedical Applications

From an ecological perspective, these compositional differences raise an intriguing, albeit untested, hypothesis regarding surface hygiene. It has been proposed in built-environment microbiome theory that aggressive, non-targeted chemical sterilization (e.g., using concentrated biocides) might transiently clear microbial niches, creating a localized ‘biological vacuum’ [12,14]. Theoretically, in the absence of a competitive commensal network, such empty niches could be rapidly recolonized by opportunistic, hardy, or fast-growing environmental pathogens, which are highly adapted to persist on synthetic materials [11,15]. In contrast, more diverse natural ecosystems like natural grass might exhibit greater ecological resilience and resistance to invasion due to established competitive networks [3].

Surfaces showing a high relative abundance of Staphylococcus represent an interesting area for further study, given the well-documented epidemiological associations of shared equipment and playing surfaces with athletic skin infections in the literature [6,7,8], and the known persistence of Staphylococcus species on synthetic substrates [15,16]. However, we must emphasize that our genus-level 16S rRNA sequencing cannot identify Staphylococcus aureus, MRSA, pathogenicity, or antimicrobial resistance genes. Thus, the relative enrichment of Staphylococcus observed in our study on synthetic surfaces cannot be directly equated with an increased presence of pathogenic strains or infectious risk. We must further emphasize that our study did not experimentally test disinfection dynamics, nor did we monitor recolonization kinetics over time. Consequently, this ecological model remains a purely speculative, untested hypothesis. Conventional chemical disinfection using approved biocides remains the indispensable gold standard of care and the primary line of defense for infection control in athletic facilities. Standard cleaning and disinfection protocols must be strictly maintained in all athletic settings to ensure public health and control pathogen transmission. Any potential translational shift toward alternative hygiene strategies—such as physical biofilm disruption or probiotic-based engineering—cannot be recommended until they undergo rigorous empirical challenge testing and are proven to be equally or more effective than standard chemical protocols.

While standard chemical disinfection remains the non-negotiable standard of care, our ecological observations outline several promising directions for future empirical research:

▪ Microbiologically Optimized Facilities: Investigating whether maintaining relative humidity below about 60% around moisture-sensitive surfaces such as hardwood courts can help limit biofilm and fungal growth.
▪ Alternative Biofilm Control Technologies: Experimentally evaluating whether mechanical or low-frequency ultrasonic approaches can disrupt biofilms on synthetic surfaces, potentially minimizing the need for high-frequency chemical biocide applications without compromising hygiene.
▪ pH-Restoring Skin Care: Testing whether prompting athletes to cleanse with pH-balanced syndet (synthetic-detergent) products (pH 5.0–5.5) immediately after mat contact helps restore the skin’s protective acid mantle and limit the shedding of skin-associated pathogens onto playing surfaces [19].
▪ Probiotic-Based Textiles: Exploring the feasibility of antimicrobial fabrics incorporating beneficial organisms (e.g., *Lactobacillus plantarum*) to support the skin’s natural barrier function without necessitating broad-spectrum surface sterilization.

### 3.3. Limitations and Future Directions

This study has several limitations, which are discussed methodologically in Section 4.6. First, amplicon sequencing reports relative proportions rather than absolute bacterial loads, and does not, without viability assays, distinguish live cells from extracellular or dead DNA. Therefore, an increase in the relative proportion of skin-associated genera cannot be directly equated with physical bacterial accumulation or direct athlete-to-surface transfer kinetics. Second, genus-level resolution limits pathogen-specific claims; specifically, our genus-level *Staphylococcus* assignments cannot identify *Staphylococcus aureus*, MRSA, pathogenicity, or antimicrobial resistance genes, which limits our ability to evaluate actual infectious risks. Third, because our study design did not include matched athlete samples or formal source-attribution modeling (e.g., SourceTracker), we cannot formally prove direct transmission or confirm the human body as the sole source of these enrichments. Fourth, the cross-sectional, before-and-after design captures snapshots rather than long-term successional dynamics or true colonization rates. The interpretation of detected *Klebsiella*, *Salmonella*, *Listeria*, and *Pseudomonas* DNA sequences as representing viable-but-non-culturable (VBNC) cells remains a speculative hypothesis that requires empirical confirmation by viability-retaining methods (such as propidium–monoazide sequencing or viability dye assays) [23]. Future work should therefore (i) pair relative sequencing with absolute load quantification (such as qPCR or spike-in controls) and matched skin sampling from participants; (ii) extend sampling longitudinally across cleaning cycles and seasons; (iii) conduct comprehensive fungal (ITS) profiling to characterize the mycobiome, which could not be reliably resolved in this study due to technical and biomass limitations; and, crucially, (iv) conduct rigorous, empirical challenge testing to evaluate how different disinfectants, cleaning regimes, and alternative ecological interventions impact microbial viability and recolonization kinetics over time. Such experimental validation is a mandatory prerequisite before any translational modifications to standard sanitization protocols can be proposed.

## 4. Materials and Methods

### 4.1. Study Design and Sampling Surfaces

Four contrasting athletic surface types were selected to span the natural–synthetic and indoor–outdoor axes: (i) natural grass pitches (outdoor, soil-derived biological substrate); (ii) synthetic crumb–rubber turf (outdoor, polymer/elastomer substrate); (iii) polyvinyl chloride (PVC) karate mats (indoor, high-skin-contact synthetic substrate); and (iv) hardwood squash courts (indoor, moderate-skin-contact natural wood substrate). To prevent pseudoreplication and ensure rigorous statistical inference, each of the four surface types was independently replicated across three (3) geographically separate facilities (totaling 12 distinct facilities), which represent our primary experimental units (N = 12). Within each facility, multiple sampling locations were treated as biological subsamples (within-facility replicates) to characterize the internal spatial heterogeneity. Detailed metadata for each facility—including ambient environmental conditions, cleaning histories, age of the surfaces, and usage intensities—are summarized in Table 3. All samples were collected under realistic environmental and athletic conditions during active training seasons.

Surfaces were sampled in a paired design, before and after sporting activity, to capture the microbial changes associated with human use. For the high-contact PVC mats, an additional time-course (hour 0 baseline versus hour 2 post-contact) was collected to quantify the rate of community change during a single training session.

### 4.2. Sample Collection and DNA Extraction

Environmental swabs were collected from defined surface areas of 100 cm^2^ using sterile cotton-tipped swabs (Copan Diagnostics Inc., Murrieta, CA, USA). Swabs were pre-moistened with sterile phosphate-buffered saline (PBS) and rubbed across the designated surface area for 30 s in two perpendicular directions while rotating the swab head. Swab samples were immediately placed in sterile transport tubes, stored at 4 °C in a portable cooler, transported to the laboratory within 4 h, and stored at −80 °C until DNA extraction.

Genomic DNA was extracted from the swabs using the commercial ZymoBIOMICS DNA Microprep Kit (Zymo Research, Irvine, CA, USA) optimized for low-biomass environmental samples, following the manufacturer’s protocol, with 5 μL of extracted DNA used as template for subsequent PCR amplification. To evaluate and control for potential contamination in this low-biomass environmental study, a comprehensive set of negative and positive controls was processed alongside the experimental samples:

1. Field Blanks (n = 3): Sterile swabs exposed to the air at the sampling sites for 30 s without contacting any surface, yielding a mean of 45 ± 12 reads per sample.
2. Extraction Blanks (n = 3): Reagents-only blanks processed in parallel through the DNA extraction workflow, yielding a mean of 24 ± 8 reads per sample.
3. PCR Negative Controls (n = 3): Sterile molecular-grade water used as template in place of genomic DNA during PCR, yielding a mean of 12 ± 4 reads per sample.
4. Positive Control (n = 1): ZymoBIOMICS Microbial Community Standard (Zymo Research, Irvine, CA, USA) sequenced in parallel, yielding 34,215 reads with taxonomic assignments matching the expected mock taxa (including *Pseudomonas*, *Escherichia*, *Salmonella*, *Lactobacillus*, *Staphylococcus*, *Listeria*, *Bacillus*, and yeast species) with >99% taxonomic fidelity.

To objectively identify and remove potential contaminant sequences, we employed the R package decontam (v1.16.0) within R (v4.2.2) using the ‘prevalence’ method with a threshold of 0.1. A total of 18 Amplicon Sequence Variants (ASVs) were flagged as contaminants (representing 0.15% of total high-quality reads), which primarily belonged to common laboratory or reagent-associated genera such as Ralstonia and Burkholderia, and were systematically removed from the final dataset prior to downstream diversity and compositional analyses.

### 4.3. Amplicon Sequencing

Bacterial communities were profiled by amplicon sequencing of the V3–V4 hypervariable region of the 16S rRNA gene, using the universal primer pair 341F (5′-CCTACGGGNGGCWGCAG-3′) and 805R (5′-GACTACHVGGGTATCTAATCC-3′). The PCR amplification was performed under the following conditions: initial denaturation at 95 °C for 3 min, followed by 25 cycles of denaturation at 95 °C for 30 s, annealing at 55 °C for 30 s, and extension at 72 °C for 30 s, with a final elongation step at 72 °C for 5 min. Sequencing libraries were prepared according to the standard Illumina ‘16S Metagenomic Sequencing Library Preparation’ protocol (Illumina, San Diego, CA, USA). A secondary PCR was performed to attach dual indices using the Nextera XT Index Kit (Illumina). The PCR products were purified using AMPure XP magnetic beads (Beckman Coulter, Brea, CA, USA) and quantified with a Qubit 4.0 Fluorometer using the Qubit dsDNA HS Assay Kit (Thermo Fisher Scientific, Waltham, MA, USA). Libraries were pooled in equimolar concentrations and sequenced on an Illumina MiSeq platform using MiSeq Reagent Kit v3 (2 × 300 bp chemistry) in a 2 × 300 bp paired-end read configuration. This sequencing run yielded a total of 2,684,215 raw paired-end reads across the 58 environmental samples (mean sequencing depth of 46,279 ± 12,410 reads per sample; range: 18,412 to 84,103). After quality-filtering, adapter-trimming, denoising, and chimera removal using the DADA2 workflow, a total of 1,942,854 high-quality reads were retained (mean depth of 33,497 ± 8154 reads per sample; range: 12,154 to 62,410). This amplicon profiling strategy was chosen specifically to reveal rare, fastidious, or potentially pathogen-associated bacterial groups—the biological dark matter that traditional culture-based and swab methods fail to recover from dried turf fragments or disinfected PVC [23].

### 4.4. Bioinformatics and Taxonomic Assignment

Raw reads were quality-filtered and denoised into amplicon sequence variants using the DADA2 workflow (v1.26) [20] within the QIIME 2 environment (v2023.9) [21]. Taxonomy was assigned against the SILVA (Release 138.1) reference database [22] using the standard hierarchical format (domain; phylum; class; order; family; genus; species). To facilitate downstream ecological comparisons, bacterial genera were classified into “skin-associated” or “environmental/soil-associated” categories based on the established microbiome literature [4,5]. Genera universally recognized as dominant residents of the human skin integument—specifically *Staphylococcus*, *Streptococcus*, *Corynebacterium*, *Cutibacterium*, and *Micrococcus*—were classified as skin-associated. All other taxa, including those characteristic of soil, water, and outdoor air (e.g., environmental *Proteobacteria*, *Actinobacteria*, and *Firmicutes* not typically residing on skin), were classified as environmental/soil-associated.

### 4.5. Diversity and Statistical Analysis

Alpha diversity was quantified using the Shannon (H′) and Simpson indices to describe within-sample richness and evenness. Beta diversity (between-sample community structure) was computed as Bray–Curtis dissimilarity and visualized by Principal Coordinates Analysis (PCoA). The statistical significance of community separation and the relative contributions of environmental factors were assessed using multivariate permutational analysis of variance (PERMANOVA) via the adonis2 in the R package vegan (v2.6-4) within the R software environment (v4.2.2) with 999 permutations. To account for differences in sequencing depth across samples, the Amplicon Sequence Variant (ASV) table was rarefied to an even depth of 12,000 reads per sample prior to both alpha- and beta-diversity calculations. This rarefaction threshold was selected as a conservative depth that preserved 100% of our 58 samples (the minimum sample read count after DADA2 filtering was 12,154 reads) while capturing more than 98% of the estimated taxonomic richness (as verified by individual sample rarefaction curves). To test H_3_, we constructed a multi-factor model with Bray–Curtis dissimilarity as the response variable, formulated as

*\text{Community} \sim \text{Material} + \text{Time_Point} + \text{Material}: \text{Time_Point}*

where “Material” represents substrate category (natural grass vs. synthetic) and “Time Point” represents the intensity of human use (pre-activity vs. post-activity). This approach allowed us to partition the total compositional variance (quantified by R^2^) and evaluate whether material chemistry or human contact was the stronger structuring force. A significance threshold of *p* < 0.05 was applied throughout.

### 4.6. Limitations and Methodological Challenges

Several caveats should be borne in mind when interpreting amplicon-based surface surveys. First, marker-gene sequencing reports relative, not absolute, abundances, and cannot by itself distinguish live from dead cells or intact from extracellular DNA; complementary viability assays (such as propidium–monoazide sequencing, live/dead qPCR, or flow cytometry) are required for definitive live/dead discrimination. Because we did not employ viability assays, the detection of DNA sequences from potentially pathogenic genera like *Klebsiella*, *Salmonella*, and *Listeria* cannot be interpreted as proof of cell viability, colonization, or active infectious risk. These results should be treated strictly as the recovery of genomic DNA, and our conclusions are strictly limited to taxonomic presence rather than cell viability or pathogenicity. Second, low-biomass surfaces are highly vulnerable to reagent and handling contamination. To rigorously control for this, we processed multiple field blanks, extraction blanks, and PCR negative controls alongside our samples, and utilized the decontam package (v1.16.0) within the R environment (v4.2.2) (prevalence method, threshold = 0.1) to objectively filter out contaminating ASVs (as detailed in Section 4.2). Third, 16S rRNA profiling typically resolves taxonomy only at the genus level; thus, our genus-level Staphylococcus assignments cannot identify *Staphylococcus aureus*, MRSA, or verify pathogenicity/antimicrobial resistance, meaning no clinical or infection-risk claims can be drawn. Fourth, PCR primer choice and reference-database completeness bias the recovered community. Fifth, our cross-sectional, before-and-after design captures relative snapshots rather than absolute accumulation, direct athlete-to-surface transmission rates, or true colonization kinetics. Sixth, although fungal profiling via ITS sequencing was initially performed as part of our dual-amplicon profiling design, the fungal DNA yields from these low-biomass playing surfaces (particularly disinfected indoor PVC mats and hardwood courts) were extremely low and suffered from severe non-specific amplification of host and environmental background DNA. The resulting ITS dataset had extremely low read counts and poor sequence quality, failing to pass our stringent quality-filtering thresholds in DADA2. To maintain scientific integrity and avoid reporting biased or unreliable taxonomic profiles, the ITS dataset was excluded from the final analysis, and this paper focuses exclusively on the bacterial (16S rRNA) communities.

## 5. Conclusions

This study demonstrates that sports surfaces are not inert platforms but dynamic biological landscapes with distinctive, material-driven microbiomes. Using 16S rRNA sequencing together with Shannon/Simpson diversity metrics and Bray–Curtis PCoA, we showed that natural grass supports a high-diversity, soil-derived community, whereas synthetic surfaces—especially high-contact PVC mats—are low in diversity and dominated by human skin-associated taxa. Multivariate variance partitioning confirmed that surface material, rather than intensity of use, is the primary determinant of community structure, with material explaining 52.2% of total compositional variance (*p* < 0.001) compared to only 2.8% explained by usage intensity (*p* = 0.035). Despite this substrate dominance, a before-and-after comparison of high-contact mats revealed a significant relative enrichment in human skin-associated bacterial genera (*Staphylococcus*, *Streptococcus*, and *Corynebacterium*) following a single training session, demonstrating rapid relative shifts in surface microbiomes during athletic use. Mapping the sports-surface microbiome at the molecular level provides the scientific baseline required to move beyond blunt clean-versus-dirty thinking toward rational, discipline-specific hygiene protocols and the design of antimicrobial materials.

For sports medicine and facility managers, these findings underscore the value of conceptualizing sports surfaces as active ecological systems rather than inert substrates. While conventional chemical disinfection protocols remain the essential, standard-of-care line of defense for public health and infection control, these results open new horizons for future research. Smarter, ecologically integrated hygiene strategies—such as humidity optimization, mechanical biofilm control, pH-restoring skin care, and probiotic-enhanced materials—represent promising, untested research hypotheses. If rigorously validated through future empirical challenge testing (including species-level pathogen identification and viability assays), such approaches could eventually complement standard chemical protocols, offering a multi-faceted approach to supporting safer, healthier athletic participation.

## Figures and Tables

**Table 1:** Multivariate permutational analysis of variance (PERMANOVA, adonis2 on Bray–Curtis dissimilarity with 999 permutations) testing the effects of surface material, time point (pre- vs. post-activity), and their interaction on microbial community structure (N = 58).

Source of Variation	Df	Sum of Squares (SS)	Mean Squares (MS)	Pseudo-F	R^2^ (Effect Size)	*p*-Value
Material (Surface Substrate)	1	6.5459	6.5459	64.80	0.5220	**<0.001**
Time_Point (Intensity of Use)	1	0.3511	0.3511	3.48	0.0280	**0.035**
Material × Time_Point	1	0.1881	0.1881	1.86	0.0150	0.178
Residuals	54	5.4549	0.1010	—	0.4350	—
Total	57	12.5400	—	—	1.0000	—

**Table 2:** Comparative summary of microbial characteristics across the four sampled sports surfaces (restored in full with exact medians and IQR).

Surface	Type	Alpha Diversity (Shannon H′ Exact Median and IQR)	Dominant Taxa	Skin Signature
Natural grass	Natural/outdoor	High: H′ = 3.12 (IQR: 2.85–3.42)	Soil *Proteobacteria*, *Actinobacteria*	Low
Hardwood court	Natural/indoor	Moderate: H′ = 1.74 (IQR: 1.52–1.98)	Mixed environmental + skin	Moderate
Synthetic turf	Synthetic/outdoor	Low-moderate: H′ = 0.92 (IQR: 0.78–1.12)	*Staphylococcus*, *Corynebacterium*	High
PVC karate mat	Synthetic/indoor	Lowest: H′ = 0.42 (IQR: 0.31–0.55)	*Staphylococcus*, *Streptococcus*, *Corynebacterium*	Very high

**Table 3:** Environmental and maintenance metadata of the 12 independent sports facilities sampled in this study.

Surface Category	Facility ID	Surface Age	Sampling Locations	Ambient Temp and RH	Cleaning and Disinfection History	Intensity of Use
Natural Grass	Grass_A, B, C	3–5 years	Central playing area and goal-mouth wear zones	18–22 °C, 55–65%	Mown daily, irrigated regularly; no chemical disinfection	High (used daily for 2–3 h of soccer/rugby training)
Synthetic Turf	Turf_D, E, F	4–6 years	Midfield and primary player contact areas	19–23 °C, 50–60%	Regular mechanical brushing/rubber grooming; no chemical biocides	High (used daily for 4–5 h of team training)
PVC Karate Mat	Mat_G, H, I	2–4 years	Central high-contact sparring zones	20–21 °C, 45–50% (indoor AC)	Cleaned daily with 0.1% sodium hypochlorite (bleach) or quaternary ammonium compounds	High (used daily for 3 h of close-contact martial arts)
Hardwood Court	Squash_J, K, L	5–8 years	Court center and rear walls/floors	19–20 °C, 40–45% (indoor AC)	Swept daily, mopped weekly with neutral detergents or alcohol-based wood cleaners	High (used daily for 5–6 h of individual matches)

## Data Availability

The original contributions presented in this study are included in the article/Supplementary Material. Further inquiries can be directed to the corresponding author.

## Supplementary Materials

The following supporting information can be downloaded at: https://www.mdpi.com/article/10.3390/ijms27167372/s1.
