# Supplementary material for: Microbial Communities Across Sports Surfaces: Exploring the Biology of Athletic Environments
Source: Int J Mol Sci. 2026 Aug 18;27(16):7372. doi: 10.3390/ijms27167372 (PMC13513609; doi:10.3390/ijms27167372)
Supplement: Supplementary file 1 [file ijms-27-07372-s001.zip › 2_Supplementary files S2. The rapid microbial shift during competition.pdf]

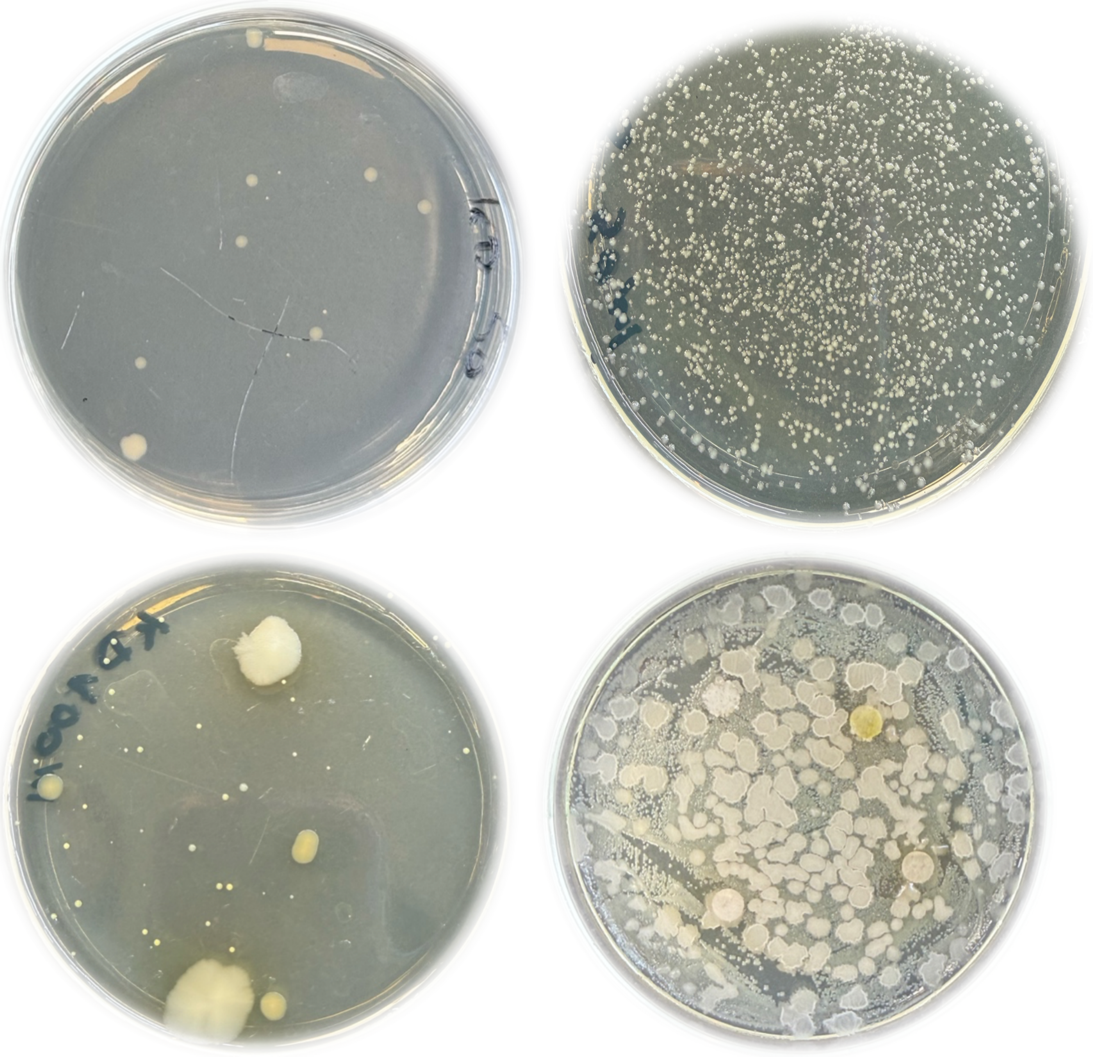

Hour 0: Baseline **Hour 2: post-contact**

Supplementary files S2. The rapid microbial shift during competition

Visual Analysis of the Cultures:
Hour 0 (Baseline): The images on the left show Petri dishes with very few, isolated microbial colonies. This represents the relatively clean state of the athletic surface (karate mats and wood corts) before use.

Hour 2 (Post-Contact): The images on the right show a dramatic increase in microbial growth, with the agar plates nearly covered in dense, diverse colonies. This demonstrates an exceptionally fast transmission of microorganisms between athletes and the environment during active training.
